# Sarcopenia, a condition shared by various diseases: can we alleviate or delay the progression?

**DOI:** 10.1007/s11739-023-03339-z

**Published:** 2023-07-25

**Authors:** Giovanni Tarantino, Gaia Sinatti, Vincenzo Citro, Silvano Jr. Santini, Clara Balsano

**Affiliations:** 1 Department of Clinical Medicine and Surgery, Federico II University Medical School of Naples, Naples, Italy; 2 Department of Life, Health and Environmental Sciences‑MESVA, School of Emergency‑Urgency Medicine, University of L’Aquila, 67100 L’Aquila, Italy; 3 Department of General Medicine, “Umberto I” Hospital, Nocera Inferiore, SA Italy; 4 Francesco Balsano Foundation, Via Giovanni Battista Martini 6, 00198 Rome, Italy

**Keywords:** Sarcopenia, Elderly, Obesity, Chronic disease, Therapy, Natural products

## Abstract

Sarcopenia is a severe condition common to various chronic diseases and it is reckoned as a major health problem. It encompasses many different molecular mechanisms that have been for a while discovered but not definitely clarified. Although sarcopenia is a disability status that leads to serious health consequences, the scarcity of suitable animal models has curtailed research addressing this disorder. Another limitation in the field of clinical investigation of sarcopenic patients is the lack of a generally accepted definition coupled with the difficulty of adopting common diagnostic criteria. In fact, both do not permit to clarify the exact prevalence rate and consequently limit physicians to establish any kind of therapeutical approach or, when possible, to adopt preventive measures. Unfortunately, there is no standardized cure, apart from doing more physical activity and embracing a balanced diet, but newly discovered substances start being considered. In this review, authors try to give an overview addressing principal pathways of sarcopenia and offer critical features of various possible interventions.

## Introduction

Sarcopenia is a harsh disorder characterized by loss of muscle mass, strength, and function in older adults, but not only, and it is associated with a variety of diseases. Peculiar symptoms include weakness, fatigue, loss of energy, balance problems, and trouble walking and standing [1]. Different animal models have been used to study sarcopenia, but the most used model is the mouse. Although the aging process in mice is similar to that in humans, aging in rodents occurs in a smaller amount of time and does not fit well with the chronicity of events that cause sarcopenia. Furthermore, important differences between rodent animal models and humans concern the different fiber type proportion (i.e. mice have a prevalence of type II fibers) and the fiber size. The difficulty of having an optimal animal model to study sarcopenia contributes to the paucity of knowledge about the molecular mechanisms that cause the onset of sarcopenia [1]. Being sarcopenia a major health problem, there is an urgent and unmet need for treatment strategies, beyond an effective prevention. Indeed, efforts are hampered by the lack of a well-accepted definition and commonly used diagnostic criteria [2]. Resulting inconsistencies in the literature negatively affect the ability to assess its clinical relevance and the treating outcomes, even though the clarification of many molecular events that have been unfolded in the past 2 decades, is currently accountable. The main aim of this review is trying to explore both the intricate downstream signaling events and the different ways in which physical activity and natural products containing polyphenols could improve not only the depletion of muscle mass but also its strength and ultimately mobility. This latter is constantly impaired in sarcopenic individuals, regardless of the underlying conditions or disease.

## Epidemiology and mechanisms

Sarcopenia in the elderly is the loss of muscle linked to ageing. It gives place to loss of muscle power, which in the end results in frailty and disability. There is an extensive evidence base on negative impact from the lack of physical activity on the onset of sarcopenia [3]. Surprisingly, the prevalence of sarcopenia varies from 8 to 40% in people over 60 years. This huge discrepancy relies on substantial differences in the age of the participants to studies, the definition of the loss of skeletal muscle mass and consequent function, and the specific tool(s) used to assess the deficit.

Among central factors affecting both the aging process and muscle loss there are increased levels of Reactive Oxygen Species (ROS), DNA damage and cell apoptosis, but mainly systemic chronic low-grade inflammation. Recent evidence points to this last mechanism as decisive contributor of the muscle mass loss, with consequent reduced strength and impaired functionality, in the sense that it involves both breakdown and synthesis of muscle proteins mediated by several signaling pathways of inflammatory nature [4]. Increased concentrations of inflammatory markers, i.e., pro-inflammatory cytokines and acute-phase proteins were considered to impact on skeletal muscle metabolism through a direct catabolic mechanism or indirect mediation, such as a decrease in growth hormones [5].

What is the role of hormonal regulation in determining or worsening the muscle loss? Deepening this aspect, we should emphasize that in aged skeletal muscle, both decreasing protein synthesis and increasing protein degradation are important events for atrophy. Since the accurate downstream signaling events of sarcopenia are still to be definitely clarified, the increasing evidence supports that skeletal muscle wasting is associated with several elements including insulin resistance, deficient nutrition, and decreased growth-promoting hormones such as Growth Hormone (GH) and Insulin-like Growth Factor-1 (IGF-1) [6], apart from the well-known elevated pro-inflammatory cytokines and physical inactivity. Also in healthy older adults, associations between higher adiponectin levels and lower muscle mass could be explained by the decrease of IGF-1 [7]. That GH acts through its receptor is established; however, many effects linked to exercise and muscle growth are believed to function “indirectly” through an increase in hepatic release of IGF-1, also in the absence of GH [8]. But the hormonal constellation involved in sarcopenia does not limit to the GH/IGF-1 axis, because hormonal roles of testosterone and estrogen in modelling muscle mass and its contractile function are evident [8].

One main concept to keep in mind is the relationship between malnutrition and sarcopenia. The term malnutrition, understood as unbalanced eating habits, includes obesity. The syndrome consists in the clinical presentation of both starvation and unbalanced diet and accelerated age-associated loss of lean body mass, strength, and/or functionality, particularly common in older adults.

Indeed, the skyrocketing prevalence of obesity observed in recent years in older individuals has led to a condition called sarcopenic obesity. This condition is a significant public health hazard that increases the risks for multiple diseases, such as type II diabetes mellitus, cardiovascular disease, hyperlipidemia, hypertension, stroke, and cancer [9].

It should be stressed that Dual-Energy X-ray Absorptiometry (DEXA) is currently reckoned as one of the most reliable methods to evaluate muscle mass, which is further adjusted for height, weight fat mass or Body Mass Index (BMI) to obtain a determination of sarcopenia status [10].

Unfortunately, many not broadly accepted definitions are present in literature. According to NHANES III, sarcopenia, being a mismatch between muscle mass and fat mass, was defined using the following cut-offs: two lower quintiles of muscle mass (fat-free mass, < 9.12 kg/m^2^ in men and < 6.53 kg/m^2^ in women) and for the obesity two highest quintiles of fat mass (> 37.16% in men and > 40.01% in women) [11]. Also in this case, the prevalence of sarcopenic obesity varies greatly according to definitions. In fact, measuring body composition by DEXA and defining sarcopenic obesity by the means of 29 definitions, the prevalence of sarcopenic obesity in 11,803 Canadians (49.6% males, 50.4% females), aged over 65, ranged from 0.1 to 85.3% in males, and from 0 to 80.4% in females. Sarcopenic obesity was frequently found associated with low handgrip strength in both males (using 14 out of 17 definitions) and females (using 21 out of 29 definitions), but with a different percentage, i.e., 82.4 and 72.4%, respectively [12]. That said, in 2022, the European Society for Clinical Nutrition and Metabolism and the European Association for the Study of Obesity launched an initiative to reach expert consensus on a definition and diagnostic criteria for sarcopenia obesity [13]. The methods, i.e., hand-grip strength or knee extensor strength or chair-stand test for the assessment of muscle strength coupled with DEXA or, as an alternative second choice, bioelectrical impedance analysis (BIA) or computerized tomography (CT) and related cut-offs are largely discussed by Donini et al. [14]. Being obesity associated with an increased risk of nonalcoholic fatty liver disease (NAFLD), participants with obesity related NAFLD had consistently less skeletal muscle mass over 12 years of follow-up. Accordingly, patients are encouraged to maintain muscle mass, and physicians are advised to give great emphasis to this aspect when managing NAFLD [15].

But what is the role of lipids excess inside muscle? Alterations of the fatty acid (FA) metabolism are evident in aging and obesity, both conditions leading up to sarcopenia, with the accumulation of lipids inside muscle cells. These lipids include diacylglycerol, lipid droplets, intramyocellular lipids, intramuscular triglycerides (IMCL) and polyunsaturated FA [16]. This muscle fat storage is reckoned as myosteatosis, deleteriously impacting on muscle strength by changing muscle fiber disorientation, and ultimately maintaining scarce mobility into later life. There is an association of myosteatosis measured using visual muscular quality map in computed tomography with NAFLD and its severity [17].

Among others, sarcopenia is one of the most common complications in advanced liver diseases, affecting one-third to two-thirds of patients with liver cirrhosis. Sarcopenia was an independent predictor of mortality in patients with cirrhosis, with a hazard ratio (HR) of 2.36 (95% CI 1.23–4.53), after adjustments for age and MELD scores [18]. The literature documented that in adults with end-stage chronic liver diseases, the prevalence rates of pre-Liver Transplantation (LT) sarcopenia, pre-LT sarcopenic obesity (SO), post-LT sarcopenia, and post-LT SO were 14–78%, 2–42%, 30–100%, and 88%, respectively [19]. Among other mechanisms of sarcopenia, much importance has been embodied by myostatin, produced and released by myocytes. This molecule inhibits muscle growth and is transcriptionally upregulated by hyperammonaemia [20]. In detail, ammonia moves in the skeletal muscle by the means of the specific transport proteins RhB and C. These glycoproteins can activate TRAF6/IKK inflammatory pathway that in the nucleus of myocytes upregulates the transcription of myostatin. Authors provided evidence that skeletal muscle results in mitochondrial dysfunction and oxidative stress due to lower NAD + /NADH ratio during hyperammonemia with reduces tricarboxylic acid cycle (TCA) intermediates and gives rise to generation of ROS [21]. Among other processes involved in sarcopenia of liver cirrhosis that should be further considered, physical activity plays a central role. Dealing with the principal association between the lack of physical activity and the loss of muscle mass and its strength, the lipid messenger phosphatidic acid is a novel mechanical mechanism involved in the activation of mammalian target of rapamycin (mTORC1) pathway that in turn regulates muscle protein synthesis and cell size [21].

We should point out that many of the features of advanced liver disease is similar to those seen in hypogonadal men, including sarcopenia. Testosterone, an important anabolic hormone with effects on muscle, is reduced in most men with cirrhosis (up to 90%) with levels falling as the liver disease worsens [22]. Men suffering from liver cirrhosis are characterized by hypoestrogenism. The increment of estrogen concentrations in cirrhosis patients results, in large part, from an increased peripheral conversion from androgens including testosterone and regulates several downstream genes and molecular targets on muscle [23]. On the other hand, cirrhotic women had markedly lower follicle stimulating hormone and sex hormone-binding-globulin levels [24].

Lastly, the role of microbiome in sarcopenia is another aspect that should be analyzed to give a more comprehensive view of the topic. First, it should be underlined that branched-chain amino acid (BCAA) supplements have proved to build muscle, decrease muscle fatigue, and alleviate muscle soreness [25]. The disruption of the gut microbiota induces the degradation of BCAA in muscle and decreases the expression of IGF-1 [26]. However, fascinating these studies may be, gut flora disruption does not tell us whether dysmicrobism is an effect, a side-effect or even the cause of the observed condition. Accordingly, malnutrition in patients with inflammatory bowel disease is also often associated with sarcopenia [27].

Along the same lines, plenty of studies in patients with chronic kidney disease have shown that sarcopenia is a prevalent condition, mainly among patients with end-stage kidney disease on hemodialysis [28]. And again, according to a systematic review including 11 studies, not a single methodology was consistent for evaluation of sarcopenia or malnutrition, although as authors clearly state, the concept of this association intuitively makes sense to clinicians [29]. Apart from the muscle fiber atrophy and weakness that both occur in respiratory muscles along with systemic skeletal muscle in ageing, known as respiratory sarcopenia, in which undernutrition plays a central role [30], there is, surprisingly, a large proportion of patients with chronic obstructive pulmonary disease (COPD) that exhibits overweight and obesity. Nevertheless, few studies examine sarcopenia and frailty in chronic respiratory disease until now. In one 2015 study, sarcopenia affected 15% of 622 outpatients with stable COPD and impaired function and deranged health status. Fortunately, pulmonary rehabilitation gave place to a reversal of the syndrome in select patients [31]. It is well established that altering oxygen delivery impacts on contracting skeletal muscle, and thus physical performance is affected [32]. At this point, it is mandatory to deeply explore this last aspect because there are relevant tools for the assessment of muscle weakness and physical performance in daily clinical practice.

Apart from the well-known actions of skeletal muscle, another important one is that such muscle functions as an endocrine organ, secreting and consequently releasing cytokines or polypeptides, known as myokines. These myokines show regulatory effects on signal transduction in skeletal muscle and the metabolism of peripheral tissues and organs. Obesity and aging cause myokine “secretion dysregulation”, speeding the sarcopenic obesity development [33].

One of the most interesting myokines, which we present now, is irisin, which protects against muscle wasting in an autocrine manner. It is generally accepted that irisin levels increase transiently following aerobic and anaerobic exercise, which could contribute to loss of fat mass [34]. Despite the clear implication of involving this myokine in muscle metabolism, the authors did not observe an association between its serum level and clinical muscle parameters in older adults, consequently not predicting risk for sarcopenia [35]. Among the myokines, low concentrations of Interleukin (IL)-15, produced by muscle in response to exercise, were associated with sarcopenia in older people [36].

It is worrying that sarcopenia that was historically recognized as an age-related disease, recently, it has been reported to be prevalent in younger patients with chronic diseases and autoimmune diseases [37]. Noteworthy is that muscle function, evaluated as both handgrip strength and short physical performance battery, was negatively associated not only with age but also with levels of free triiodothyronine (FT3). What is more, higher FT3 concentration within normal range was correlated to muscle mass and muscle function in elder subjects [38].

Among other diseases accompanied by sarcopenia, we should conclude this first part of our review highlighting the key-role of osteoarthritis that plays a main role in inducing loss of muscle mass, favoring the scarce mobility [39].

## Therapeutical approaches

Exploratory therapeutic approaches to sarcopenia should address the following aspects: inflammatory responses, metabolic alterations, oxidative stress, apoptosis/senescence and myosteatosis. Regarding measures that primarily address chronic low-grade inflammation and oxidative stress, we will once again mention the main role of physical exercise. Large population-based cohort studies demonstrate an inverse association between markers of systemic inflammation and physical activity [40]. The positive effects of exercise on inflammation consist in improved endothelial function through increased eNOS protein expression and phosphorylation [41] and result from modulation of intracellular signaling pathways involving ROS and myokines [42]. Many clinical practice guidelines strongly recommend increased physical activity as a useful intervention for patients with sarcopenia. Although it is difficult to define a sequence of cause-and-effect events when muscle interacts with exercise and to accurately predict whether some patients with sarcopenia will become responsive to physical exercise, these specific issues have been tested experimentally with non-unique results. Indeed, in a general review in 2022, Y. Shen et al. assessed the methodological quality and evidence of systematic reviews for all associations between physical activity and sarcopenia. The conclusions maintain the current recommendations on exercise, although posing a note of caution about elderly patients with sarcopenia [43]. In the same year, H. Wang et al. in a meta-analysis involving 23 studies and 1252 elderlies with sarcopenia, showed that physical exercise can effectively improve muscle function and physical performance in the elderly with sarcopenia, but has limited effects on upper limb muscle mass [44]. Furthermore, to prevent sarcopenia in the elderly population, resistance training is highly recommended over aerobic exercise, which does not appear to have demonstrated the same benefits [44]. It is impossible to draw definitive conclusions from these latest studies, but it is important to investigate the role played by physical activity as a promoter of positive mental health and the perception of well-being, which in turn leads to increased mobility, which is often and for various reasons decreased in the elderly [45]. As previously discussed, one of the main triggers of the imbalance between protein synthesis and degradation that leads to muscle atrophy can be considered oxidative stress, characterized by increased production of ROS [46]. In this sense, the use of antioxidants compounds to reduce the damage associated with oxidative stress has proved effective against skeletal muscle atrophy [46]. As natural compounds with proven antioxidant activity, polyphenols play an important role by counteracting the pro-oxidant enzymes NOX-2 and NOX-4 [47], and with an anti-inflammatory effect that attenuates the activation of the NF-κB [48]. Polyphenols not only inactivate NF-κB, but also modulate the mitogen-activated protein kinase (MAPK) and arachidonic acid pathways. Polyphenolic compounds inhibit PI3K/Akt, IKK/JNK inhibitor, mTORC1, and the JAK/STAT signaling pathway. Finally, they can suppress the expression of pro-inflammatory genes and the expression of NOX-1, with a concomitant decrease in ROS generation [49].

We present some recent data from the literature on natural products containing polyphenols (Table 1). Few reports describe the beneficial effects of gallic acid (GA) and ellagic acid (EA) on skeletal muscle. However, K. B. Hong et al. (2020) demonstrated the antioxidant and anti-inflammatory role of tannase-converted green tea extract with a high GA and EA content on myotube as a prevention of skeletal muscle function decline due to oxidative stress [50]. Again, Felice et al. (2021) explored the effect of tomato on a primary human skeletal muscle cell after inducing sarcopenia in vitro with dexamethasone [51].

**Table 1:** Effects of polyphenols

Authors	Year	Polyphenol	Type of study	Effects
Hong KB et al. [50]	2022	Tannase converted-green tea	In vitro	The levels of AMP-activated protein kinase-α (AMPKα) and muscle RING-finger protein-1 (MuRF-1)
Felice F et al. [51]	2020	Gallic acid	In vitro	↓Atrogin-1, MuRF1 two muscle-specific ubiquitin ligases linked to muscle atrophy
Hour TC et al. [53]	2022	Quercetin	In vitro	↑p-IGF-1R; regulating the ITGB1 signaling pathway and activating phosphorylation of FAK and paxillin
Funakoshi T et al. [54]	2017	Quercetin	In vitro and in vitro	↑PPAR-g expression of FABP4
Park SH et al. [55]	2020	Quercetin	In vivo and in vitro	The gene expression of atrogin-1 and MuRF1 and phosphorylation of AMPK
Suzuki K et al. [57]	2020	Theaflavins	In vivo	Anabolic Akt/mTOR pathway; phosphorylation of 4EBP-1
Liu C et al. [58]	2022	Theaflavins	In vivo and in vitro	Expression of IL-1β, IL-6 and TNF-α by regulating the TLR4/MyD88/NF-κB signalling pathway
Aizawa T et al. [59]	2017	Theaflavins	Pilot study	Theaflavin had a beneficial effect on body fat and muscle
Fujiwara Y et al. [60]	2017	Oleuropein	RCT	↑Traslocation of GLUT4 by activation of adenosine monophosphate-activated protein kinase (AMPK)
Razaei S [61]	2018	Olive oil	RCT	↓Body fat mass, not reduced the skeletal mass
Santini SJ et al. [62]	2020	Oleuropein	In vivo and in vitro	↓Fat in HepG2 cells ↓IL1-α and G-CSF ↑SOD2 cytosol expression
Santini SJ et al. [63]	2022	Oleuropein	In vivo and in vitro	↓fat in HepG2 cells ↓copper content of FA-HepG2 Less body and liver weight; Improved the level of transaminases and lipid profile
Porcu C et al. [64]	2018	Oleuropein	In vivo and in vitro	↑Activation of Akt/ULK1 pathway

Quercetin, a natural flavonoid, has an anti-inflammatory and antioxidant effect in vivo and in vitro, and has an impact on serum cholesterol. Interestingly, it has also proven to have protective effects against muscle atrophy in cachexia and obesity [52]. Hour et al. (2022) explored the effect of quercetin on mouse skeletal muscle regeneration, focusing on the mechanisms of induction of myogenic differentiation, migration, and differentiation. Compared to the control, quercetin treatment, through activation of p-IGF-1R, produced more myotubes with a dose-dependent system [53]. Funakoshi et al. (2018) demonstrated that quercetin can also limit the ectopic lipid accumulation as observed in aged muscles by inhibiting the differentiation of primary muscle satellite cells (mSCs) into adipocytes. Indeed, quercetin supplementation counteracts oxidative damage in atrophied skeletal muscle by reducing lipid peroxidation, acting on a key transcriptional regulator for adipogenesis, peroxisome proliferator-activated receptor-gamma (PPAR-gamma) and regulating the expression of a lipid transporter, FA-binding protein 4 (FABP4) [54]. That quercetin can be an effective supplement or therapeutic agent to prevent or treat sarcopenia was demonstrated by Park et al. (2020) who found out that quercetin 3-O-beta-glucuronide, a major active component of the lotus leaf, improved muscle wasting in dexamethasone-induced muscle atrophy in mice [55].

Theaflavins are polyphenols contained in high concentrations in black tea, known for their ability to reduce plasma low-density lipoprotein-cholesterol (LDL) levels and blood pressure, leading to a significant reduction in cardiovascular risk [56]. Suzuki et al. (2021) examined the effect of theaflavins on model mice of disuse muscle atrophy by suspension of the hind limbs. On histological examination, the size of the myotubes was significantly smaller in the suspension group than in the ground group. This difference was significantly reduced by theaflavin treatment, suggesting that repeated intake of theaflavin may inhibit the progression of disuse muscle atrophy through modulation of protein metabolism [57]. Recent evidence demonstrates the beneficial effect of theaflavins even in the presence of inflammatory infiltration in muscle. In fact, Liu et al. (2022) performed in vitro and in vivo models of muscle inflammation and claim that theaflavins reduce the expression of inflammatory factors like IL-1β, IL-6 and TNF-α [58]. Aizawa et al. (2017) also studied the beneficial effect of theaflavins on body fat and muscle in healthy individuals in a RCT. Compared to the placebo and catechin groups, the theaflavin-treated group demonstrated a reduction in subcutaneous fat levels, resulting in improved body composition [59].

Oleuropein (Ole), one of the main phenolic compounds in olives, has proven effective in reducing insulin resistance in muscle cells by promoting the translation of glucose transporter 4 through the activation of 5' AMP-activated protein kinase (AMPK), independently of insulin signals, with beneficial effects on body mass composition [60]. Razaei et al. (2019) examined the effect of olive oil on the severity of fatty liver in patients with NAFLD, who often present with sarcopenia, and showed that, compared to the control group, changes in the degree of fatty liver damage and skeletal muscle mass were greater in subjects in the olive oil group. In details, fat mass and body fat percentage decreased significantly in the olive oil group, with a difference between the groups in skeletal muscle mass [61]. Finally, regarding Ole’s antioxidant antiglycative and anti-inflammatory capabilities, Santini et al. (2020 and 2022) demonstrated that it can improve the pro-inflammatory and antioxidant ‘defence state’ in a mouse model of NAFLD [62, 63]. Furthermore, the oral administration of Ole in C57BL/6 J mice on an unhealthy diet induced the activation of autophagy characterized by AMPK-dependent phosphorylation of ULK1 at Ser555, regardless of sex [64], (Table 1). Interestingly, in a rare animal model study of sarcopenia obesity, Codonopsis lanceolata, also known as bastard ginseng, administered to C57BL/6 mice fed a high-fat diet, attenuated muscle atrophy by inhibiting lipid accumulation in skeletal muscle (myosteatosis) through restoration of the PI3K/Akt pathway and impaired lipid metabolism [65].

Recently, independently of polyphenols, some other natural compounds, such as ursolic acid, enhance insulin/IGF-I signaling in skeletal muscle and inhibit skeletal muscle atrophy-associated mRNA expression [66], tanshinone IIA, which stimulates C2C12 myotube hypertrophy through an estrogen receptor-mediated mechanism [67], tomatidine, which stimulates mTORC1 signaling and anabolism, leading to the accumulation of proteins and mitochondria and, ultimately, cell growth [68] and, finally, magnoflorin, which prevents skeletal muscle atrophy via the Akt/mTOR/FoxO signaling pathway [69], are widely known to promote muscle mass and strength.

Although these studies on natural products cannot provide a causal link, the ‘not-so-eerie’ correlation between these products and muscle metabolism undoubtedly deserves to be taken into account, especially when assessing the frailty associated with loss of muscle mass in the elderly or those with advanced chronic liver disease. These latter stratified patients could benefit from early supplementation with palliative care and in this sense, natural products could be useful.

It is widely accepted that a healthy lifestyle and nutritional habits are essential for maintaining a healthy state. As already mentioned, in addition to physical exercise, GH, IGF-I, testosterone and estradiol, adrenal-derived dehydroepiandrosterone, thyroid hormones and vitamin D are involved in sarcopenia. Therefore, could hormone replacement be helpful in trying to mitigate sarcopenia? First, we should exclude adrenal steroids. Indeed, higher levels of salivary cortisol at 11 a.m. and 8 p.m. in postmenopausal women have been found to be associated with sarcopenia [70]. However, looking at the results of a systematic review comprising 23 RCTs, i.e., 4 studies focusing on protein as a dietary supplement, 7 on essential amino acids, 6 on creatine, 4 on dehydro-epiandrosterone and finally 2 on β-hydroxy β-methylbutyrate, it appears that the effects of these supplements on muscle mass, function and physical performance are rather limited [71]. Restoration of normal albumin concentrations, associated with dietary supplementation of BCAA, is correlated with a reduction of fat accumulation in skeletal muscle, preservation of muscle mass and improved glucose sensitivity in patients with liver cirrhosis [72]. Interestingly, an ancillary analysis of two RCTs, treatment of subclinical hypothyroidism had no significant effect on function, strength and muscle mass in subjects aged 65 years and older [73]. Compared to placebo, testosterone replacement (7.5 g testosterone gel at 1% per day) in elder men with low to moderate–normal testosterone levels for 3 years was associated with modest but significantly greater improvements in muscle mass [74]. L-carnitine is the only natural steroid approved by the Food and Drug Administration (FDA) for short-term use in healthy adults over the age of 18 years to improve athletic performance [75]. Given its central role in FA oxidation and energy metabolism, its supplementation decreased oxidative stress and improved glucose and lipid metabolism through the regulation of PPAR-gamma, GLUT-2 and GLUT-4 mRNA expression in rat muscle [76].

The treatment of frailty and sarcopenia overlap. Therefore, adequate protein and vitamin D (Vit-D) supplementation may be a promising therapeutic approach, in addition to exercise. Supplementation with whey protein, essential amino acids and Vit-D, combined with physical exercise, increases muscle mass and strength, but also improves other aspects that contribute to the well-being of sarcopenic elderly [77].

Growing evidence points to the importance of the microbiota in the gut–brain–muscle axis [78]. This is characterized by the involvement of gut flora that has an impact on skeletal muscle energy and muscle fiber conversion through its metabolites [78]. In a recent RCT of 60 over 65 years old patients, the administration of prebiotics compared to placebo significantly improved muscle function, exhaustion and grip strength [79]. Probiotics capable of limiting sarcopenia in mice are mainly lactic acid bacteria and bifidobacterial. However, the same bacteria tested in humans, due to the variability of populations and the difficulty of accurately measuring muscle mass and function, have not allowed to determine which specific strains should be used to improve physical performance [80].

## Conclusions

Sarcopenia is a disorder common to various chronic diseases, encompassing many fascinating molecular mechanisms that are tightly intertwined with themselves. There is a huge debate on how physicians should therapeutically approach this condition to obtain a reduction/recovery of muscle loss. Continuing efforts are needed to try to ameliorate patients’ mobility. Unfortunately, there is no standardized therapy, apart from increasing physical activity and adopting a balanced diet, but promising remedies and novel substances appear on the scene.

## Future direction

Slowing the progression of sarcopenia consists in identifying underlying causes and subsequently correcting them, when possible. The necessity of resistance training should be emphasized, and care givers ought to ensure an adequate food intake to elderly and suffering people. Muscle satellite cells (mSC) are essential in preserving muscle health until old age. These specific stem cells are fundamental in skeletal muscle regeneration, and it has been suggested that a reduction and/or dysregulation of the mSC pool could speed the loss of muscle mass that is a frequent finding in the elderly.

## Data Availability

Not applicable.
